# A registry study of oral health problems and preventive interventions among older persons receiving municipal healthcare – PROSENIOR

**DOI:** 10.1002/nop2.1318

**Published:** 2022-08-14

**Authors:** Malin Axelsson, Christel Bahtsevani, Merita Neziraj, Karin Persson, Christine Kumlien

**Affiliations:** ^1^ Department of Care Science, Faculty of Health and Society Malmö University Malmö Sweden; ^2^ Department of Cardio‐Thoracic and Vascular Surgery Skåne University Hospital Malmö Sweden

**Keywords:** nursing, oral health, prevention, risk assessment, Senior Alert

## Abstract

**Aim:**

The aim was to identify planned and completed preventive interventions among older persons with oral health problems receiving municipal health care. A further aim was to determine the correspondence between oral health problems and planned preventive interventions among older persons with oral health problems receiving municipal health care.

**Design:**

Cross‐sectional register study.

**Methods:**

Oral health data from the Swedish national quality registry, Senior Alert, were extracted for 4,024 older persons (≥65 years) receiving municipal health care in a county in Southern Sweden. Data were statistically analysed.

**Results:**

A large majority of older persons (97.4%) with assessed oral health problems had at least one planned preventive intervention, and approximately three quarters of the planned interventions were completed. There seemed to be a mismatch between type of oral health problems and preventive interventions as not all older persons had a planned preventive intervention related to their specific oral health problem.

## 1 INTRODUCTION

Oral health is known to impact on the quality of life in older persons (Lindmark et al., 2020; McGrath & Bedi, 1991; van de Rijt et al., 2021), and oral health is experienced as central for the sense of self‐esteem and thus is perceived as a priority by older persons (McKenzie‐Green et al., 2009). Importantly, poor oral health is associated with malnutrition (Lindroos et al., 2019; van Lancker et al., 2012) and is a risk factor for aspiration pneumonia among older persons (van der Maarel‐Wierink et al., 2011), which in turn increases the risk for death (Welte et al., 2012). However, a recent review (Manger et al., 2017) shows strong evidence that the incidence of pneumonia can be reduced by regular oral hygiene interventions, that is through the use of chlorhexidine in mouthwash or gel among older persons. Another review (van der Maarel‐Wierink et al., 2013) concludes that tooth brushing after each meal, cleaning dentures once a day and professional oral health care once a week reduce the incidence of aspiration pneumonia among frail older persons.

## 2 BACKGROUND

It is to be noted that poor oral health among older persons is common both internationally and nationally, and a systematic review (Wong et al., 2019) reported that a majority of older persons had problems with oral cleanliness and health. Moreover, the prevalence of poor oral health in Italian nursing homes has been estimated to be approximately 44% and even higher among those with cognitive impairment (54%) and those with higher dependency on assistance (56%) (Chiesi et al., 2019). In Norwegian nursing homes, 40% had poor oral health (Willumsen et al., 2012), and in Swedish nursing homes, 42% had oral health problems and men had more oral health problems than women (Bellander, Andersson, Nordvall, et al., 2021). Importantly, the prevalence of oral health problems differs between types of housing, where oral health problems are more common in home health care than in nursing homes (Czwikla et al., 2021). A recent study (Neziraj, Hellman, et al., 2021), conducted in southern Sweden, shows that the prevalence of the risk for poor oral health was approximately 34% among older persons receiving municipal health care, that is community care service of older persons. The care of older people is in Sweden primarily a municipal responsibility and mainly financed by taxes (Swedish Institute, 2021). Although the prevalence of the risk for poor oral health varied in different types of housing – 31% among older persons in short‐term nursing care, 28% among older persons living in their own homes and receiving municipal health care, and 34% in nursing homes – the prevalence of the risk for poor oral health was greatest among older persons in dementia care units (42%) (Neziraj, Hellman, et al., 2021).

Essentially, increasing dependence on others in everyday life and decreasing cognitive ability among older persons leads to poorer oral health status and increased need for assistance with oral care (Zenthöfer et al., 2014). Although promoting oral health is a responsibility for nurses as being one of the fundamentals of nursing care (Kitson et al., 2010), assisting older persons with their oral health is often overlooked by nurses (Costello & Coyne, 2008; Ek et al., 2018). Reasons for this oversight have been related to lack of knowledge, education and training in providing oral care, time constraints, general dislike of oral care among the nursing staff (Wårdh et al., 2012). Other reasons have been related to working experience, that is nurses with shorter experience seem less likely to provide oral care (Edman & Wårdh, 2022). A recent review (Oda et al., 2022) found that barriers for nurses to provide oral care were lack of clear guidelines and protocol for the provision of oral care. Another reason is older persons' resistance to receiving help with oral care (Hoben et al., 2017; Oda et al., 2022). Altogether, this can be reflected in a large unmet need for oral care among older persons, which has been shown in a Swedish study by Forsell et al. (2009) reporting that only 6.9% of those in need of assistance with oral care actually received it. There is, as far as we know, nothing to suggest that this problem has changed for the better over time.

Despite the fact that oral health problems are common among older persons worldwide and constitute a risk for morbidity and mortality, oral health care seems to be a nursing element with room for improvement, and there is a need for increased knowledge about preventive interventions especially in elderly care in Sweden.

Consequently, the aim of the current study was to identify planned and completed preventive interventions among older persons with oral health problems receiving municipal health care. A further aim was to determine the correspondence between oral health problems and planned preventive interventions among older persons with oral health problems receiving municipal health care.

## 3 THE STUDY

### 3.1 Design

The current study is part of the research project PROSENIOR, which focuses on the prevention of common risk factors such as poor oral health among older persons receiving municipal care (https://mau.se/en/research/projects/prosenior/). The design was a cross‐sectional register study based on oral health data extracted from the Swedish national quality registry called Senior Alert, which is one of the largest and most frequently used quality registries in Sweden. Senior Alert is a well‐integrated tool for the prevention of pressure ulcers, falls, malnutrition, and poor oral health among older persons (≥65 years). Prior to registration, older persons are informed about the registry by their healthcare providers, and they give their oral consent for registration. Thereafter, healthcare providers register data from the conducted risk assessments and planned and completed preventive interventions. The intention of the registry is to systemize the preventive care work, reduce risks and improve patient safety (Edvinsson et al., 2015; Senior Alert, 2021). The current study followed the STROBE guidelines (von Elm et al., 2008).

### 3.2 Method

#### 3.2.1 Study population

The current study was based on 4,024 older persons (≥65 years) receiving municipal health care in a county in Southern Sweden and whose oral health problems and associated preventive interventions were registered in Senior Alert.

#### 3.2.2 Data extraction

Oral health data from Senior Alert were extracted for the period August 1, 2018, to July 31, 2019. The extracted data constituted the first reported risk assessment and associated planned and completed preventive interventions based on the Revised Oral Assessment Guide‐Jönköping (ROAG‐J) registered during this time period. The total sample consisted of 12,518 persons aged ≥65 years, which were registered in Senior Alert in the selected county in southern Sweden during the set time period but oral health data including associated preventive interventions were only available for 4,024 older persons.

#### 3.2.3 Instrument

The Revised Oral Assessment Guide (ROAG), which is used for oral assessments in older persons, has shown both good inter‐rater reliability (Andersson et al., 2002) and reproducibility (Ribeiro et al., 2014). In Senior Alert, a somewhat modified version, the ROAG‐J, is used to assess risks for poor oral health. ROAG‐J also includes recommendations about what interventions to undertake for the different types and causes of oral health problems. The ROAG‐J evaluates oral health by assessing the condition of the following nine categories: Voice, Lips, Mucous membranes, Tongue, Gums, Teeth, Dentures, Saliva and Swallowing. Each category is measured on a scale of 0–3, where scores of 0 and 1 indicate no risk, while scores of 2 and 3 indicate risk for oral health problems. According to ROAG‐J, the nursing staff should plan and implement a preventive intervention when the older persons score a 2, while a dentist should be consulted and offered when the older persons score a 3 on any assessment (Senior Alert, 2021).

#### 3.2.4 Definitions

The type of housing was defined according to Neziraj, Hellman, et al. (2021):

*Short‐term nursing care*: a shorter stay facility for older persons at special municipal residential care homes that offer rehabilitation, aftercare, diagnosis and assessment of needs.

*Home health care*: receiving health care in one's own home.

*Nursing home care*: receiving municipal health care in residential care homes.

*Dementia home care*: receiving municipal health care in residential care homes for older persons with dementia diagnosis.

### 3.3 Analysis

SPSS version 26 (IBM Corp) was used for statistical analyses. Descriptive statistics (frequencies, percentages, means and standard deviation [SD]) were used to describe the study population. Chi‐square tests were used to test differences in proportions between men and women. *p*‐values < .05 were considered statistically significant. Missing data were handled according to the listwise deletion, that is in analyses including planned interventions the complete cases were used (Kang, 2013).

### 3.4 Ethics

Data in the current study were used after approval from Senior Alert and the Regional Ethical Review Board at Lund University in Sweden (DNR 2015‐484). The quality registry Senior Alert is one of 100 registries in Sweden used to develop the quality of care and offers a unique opportunity for performing research in health care. The use of register data is meticulously controlled in accordance with Swedish law under The Swedish Code of Statutes (SFS), Patient Data Act (2008:355). Senior Alert must ensure that the information is formulated in such way that it is clear to the older person that the registry data may be used in research and that the older person has the legal right to have their personal information removed from Senior Alert at any time (SFS, 2008:355).

## 4 RESULTS

The total sample consisted of 4,024 older persons at risk for oral health problems (64.1% women) with a mean age of 85.4 (SD 8.0) years and a majority (67.5%) living in nursing homes (Table 1). The proportion of any planned intervention for oral health problems was 97.4% in the total sample and was similar in both women and men. Approximately 98% of the older persons in short‐term nursing care had a planned intervention and 94.6% in home health care. Regarding planned interventions that were registered as completed for oral health problems, the highest proportion was found in dementia care units (80.3%) and the lowest in home health care (63.5%) (Table 2).

**TABLE 1:** Background characteristics of the study participants with risk for poor oral health (*N* = 4,024)

	Total sample	Women	Men
	*N* (%)	*N* (%)	*N* (%)
Sex		2,578 (64.1)	1,446 (35.9)
Type of housing			
Dementia home care	727 (18.1)	486 (18.9)	241 (16.7)
Home health care	432 (10.7)	259 (10.0)	173 (12.0)
Short‐term nursing care	147 (3.7)	65 (2.5)	82 (5.7)
Nursing home care	2,718 (67.5)	1,768 (68.6)	950 (65.7)
Age (mean, standard deviation)	85.4 (8.0)	86.9 (7.5)	82.8 (8.0)

**TABLE 2:** Distribution of any planned and completed interventions for oral health problems among older people presented for the total sample and by sex and type of housing

	Total sample, *N* = 3,761	Women, *N* = 2,400	Men, *N* = 1,361	Dementia home care, *N* = 697	Home health care, *N* = 392	Short‐term nursing care, *N* = 142	Nursing home care, *N* = 2,530
	*N* (%)	*N* (%)	*N* (%)	*N* (%)	*N* (%)	*N* (%)	*N* (%)
Any planned intervention for oral health problems^a^	3,664 (97.4)	2,331 (97.1)	1,333 (92.2)	683 (98.0)	371 (94.6)	139 (97.9)	2,471 (97.7)

	Total sample, *N* = 3,452	Women, *N* = 2,204	Men, *N* = 1,248	Dementia home care, *N* = 656	Home health care, *N* = 326	Short‐term nursing care *N* = 129	Nursing home care, *N* = 2,341
	*N* (%)	*N* (%)	*N* (%)	*N* (%)	*N* (%)	*N* (%)	*N* (%)
Any completed intervention for oral health problems^b^	2,560 (74.2)	1,642 (74.5)	918 (73.6)	527 (80.3)	207 (63.5)	84 (65.1)	1,742 (74.4)

^a^ Missing *n* = 263.

^b^ Missing *n* = 572.

### 4.1 Oral health problems

Oral health problems related to the *Teeth* in terms of *Coating or food debris locally* were reported among 43.4% of the older persons, and *Coating, food debris generally and broken teeth* was reported among 16.5%. The proportion of oral health problems related to the *Gums* in terms of *Swollen and reddened* was 22.2% in the total sample and 23.7% in the women and 19.5% in the men. The proportion of *Minor swallowing problems* was 18.5% in the total sample with similar distributions among men and women (Table 3).

**TABLE 3:** Oral health problems according to the revised Oral assessment guide‐Jönköping (ROAG‐J), in which scores of 2 and 3 indicate oral health problems

	Total sample, *N* = 4,024	Women, *N* = 2,578	Men, *N* = 1,446
	*N* (%)	*N* (%)	*N* (%)
Voice			
0 Not applicable	149 (3.7)	86 (3.3)	63 (4.4)
1 Normal	3,213 (79.8)	2,062 (80.0)	1,151 (79.6)
2 Dry, hoarse, cracking	471 (11.7)	304 (11.8)	167 (11.5)
3 Difficult to speak	191 (4.7)	126 (4.9)	65 (4.5)
Lips			
1 smooth, light red, moist	3,502 (87.0)	2,208 (85.6)	1,294 (89.5)
2 Dry, cracked, sore corners of the mouth	519 (12.9)	368 (14.3)	151 (10.4)
3 Ulcerated, bleeding	3 (0.1)	2 (0.1)	1 (0.1)
Mucous membrane			
1 Bright red, moist	3,314 (82.4)	2,118 (82.2)	1,196 (82.7)
2 Red, dry, or areas of discoloration, coating.	673 (16.7)	438 (17.0)	235 (16.3)
3 Wounds with or without bleeding, blisters	37 (0.9)	22 (0.9)	15 (1.0)
Tongue			
1 Pink, moist with papillae	3,364 (83.6)	2,161 (83.8)	1,203 (83.2)
2 No papillae, red, dry, coating	648 (16.1)	405 (15.7)	243 (16.8)
3 Ulcers with or without bleeding, blistering	12 (0.3)	12 (0.5)	0 (0.0)
Gums			
0 No gums, only mucosa	188 (4.7)	113 (4.4)	75 (5.2)
1 Light red and solid	2,808 (69.8)	1,773 (68.8)	1,035 (71.6)
2 Swollen and reddened	894 (22.2)	612 (23.7)	282 (19.5)
3 Spontaneous bleeding	134 (3.3)	80 (3.1)	54 (3.7)
Teeth			
0 No natural teeth	492 (12.2)	305 (11.8)	187 (12.9)
1 Clean, no visible coating or food debris	1,124 (27.9)	768 (29.8)	356 (24.6)
2 Coating or food debris locally	1,745 (43.4)	1,120 (43.4)	625 (43.2)
3 Coating, food debris generally, broken teeth	663 (16.5)	385 (14.9)	278 (19.2)
Dentures			
0 No prosthetics	2,769 (68.8)	1,750 (67.9)	1,019 (70.5)
1 Clean, functioning	669 (16.6)	464 (18.0)	205 (14.2)
2 Coating or food debris	248 (6.2)	157 (6.1)	91 (6.3)
3 Not used or malfunctioning	338 (8.4)	207 (8.0)	131 (9.1)
Saliva			
1 Runs freely	3,259 (81.0)	2,065 (80.1)	1,194 (82.6)
2 Runs sluggishly	731 (18.2)	490 (19.0)	241 (16.7)
3 Does not run at all	34 (0.8)	23 (0.9)	11 (0.8)
Swallowing related to pain and dry mouth			
0 Not applicable	379 (9.4)	234 (9.1)	145 (10.0)
1 Unimpeded swallowing	2,609 (64.8)	1,675 (65.0)	934 (64.6)
2 Minor swallowing problems	745 (18.5)	485 (18.8)	260 (18.0)
3 Pronounced swallowing problems	291 (7.2)	184 (7.1)	107 (7.4)

### 4.2 Causes for oral health problems

Of the investigated causes, *Impaired ability to understand information and instructions* was reported among 39.4%. *Sore mouth at or between meals* was significantly more common among women than men at 4.7% versus 3.2%. (Table 4).

**TABLE 4:** Causes for oral health problems

	Total sample, *N* = 3,682^a^	Women, *N* = 2,357	Men, *N* = 1,325	*p*‐values comparing women and men^b^
	*N* (%)	*N* (%)	*N* (%)	
Sore mouth at or between meals	153 (4.2)	111 (4.7)	42 (3.2)	**.025**
Impaired hand/arm function or impaired general condition that complicates oral care	723 (19.6)	466 (19.8)	257 (19.4)	.784
Impaired function in the mouth and facial muscles	273 (7.4)	177 (7.5)	96 (7.2)	.769
Impaired sensation in the mouth	101 (2.7)	58 (2.5)	43 (3.2)	.162
Impaired ability to understand information and instructions	1,449 (39.4)	933 (39.6)	516 (38.9)	.702
Short intervals between meals	242 (6.6)	149 (6.3)	93 (7.0)	.413

^a^ Missing *n* = 342.

^b^ Chi‐square test.

Bold value indicates significant difference.

### 4.3 Planned preventive interventions

The most frequent planned preventive interventions were *Tooth brushing* (planned for 56.2%) and *Moisturizing the mouth* (planned for 22.2%), while *Local pain relief* of the lips, mucous membranes and tongue were rarely planned. *Consultation with a dentist* when scoring 3 on any of the oral health assessments was planned for 9.7%, while 7.5% *Resisted all preventive measures* (Table 5).

**TABLE 5:** Planned interventions grouped according to the revised Oral assessment guide‐Jönköping (ROAG‐J), *n* = 3,761 (263 missing)

Interventions	Planned, *N* (%)
Voice	
Moisturizing the mouth	835 (22.2)
Lips	
Lubricating the lips	811 (21.6)
Local pain relief	2 (0.1)
Mucous membrane	
Moisturizing	636 (16.9)
Removal of scabs	61 (1.6)
Local pain relief	5 (0.1)
Tongue	
Cleaning of the tongue	286 (7.6)
Moisturizing of the tongue	515 (13.7)
Local pain relief of the tongue	5 (0.1)
Gums, teeth, implants	
Information/training in oral health	609 (16.2)
Toothbrushing	2,113 (56.2)
Cleaning between teeth	796 (21.2)
Moisturizing	102 (2.7)
Fluoride	343 (9.1)
Extra oral care	42 (1.1)
Dentures	
Information/training in cleaning dentures	130 (3.5)
Assistance with cleaning dentures	465 (12.4)
Cleaning of remaining teeth and mucous membrane	218 (5.8)
Saliva	
Moisturizing	631 (16.8)
Swallowing related to pain and dry mouth	
Ease pain by moisturizing	361 (9.6)
Other intervention – oral health	484 (12.9)
Person resists all preventive measures	281 (7.5)
Oral health palliative care	46 (1.2)
Consultation with dentist when scoring 3 on any assessment	363 (9.7)

### 4.4 Correspondence between type of oral health problem and planned preventive interventions

**FIGURE 1:**
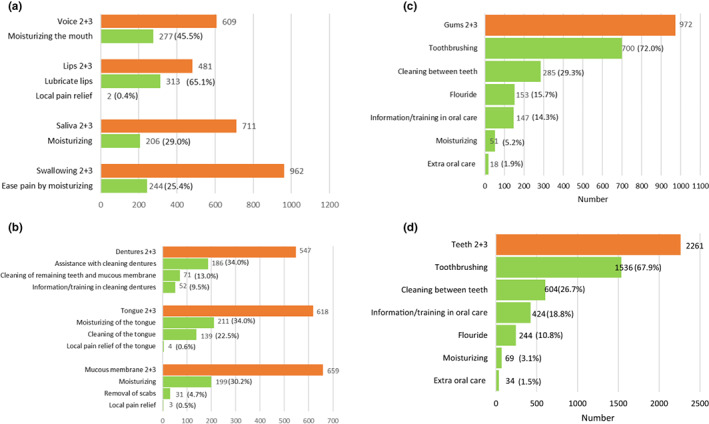
(a) The correspondence between oral health problems (orange bars) related to *voice* (*n* = 609, missing *n* = 53), *lips* (*n* = 481, missing *n* = 41), *saliva* (*n* = 711, missing *n* = 54), and *swallowing related to pain and dry mouth* (*n* = 962, missing *n* = 74) and their respective planned preventive interventions (green bars) according to the ROAG‐J. (b) The correspondence between oral health problems (orange bars) related to *dentures* (*n* = 547, missing *n* = 39), *tongue* (*n* = 618, missing *n* = 42) and *mucous membrane* (*n* = 659, missing *n* = 51) and their respective planned preventive interventions (green bars) according to the ROAG‐J. (c) The correspondence between oral health problems (orange bar) related to *gums* (*n* = 972, missing *n* = 56) and planned preventive interventions (green bars) according to the ROAG‐J. (d) The correspondence between oral health problems (orange bar) related to *teeth* (*n* = 2,261, missing *n* = 147) and planned preventive interventions (green bars) according to the ROAG‐J

Figure 1a–d illustrate the correspondence between the types of oral health problems and planned preventive interventions among older persons with different oral health problems according to the ROAG‐J. Fewer than half of the older persons with *Problems related to the voice, lips and saliva* and *Swallowing problems related to pain and dry mouth* had planned interventions related to these oral health problems, with the exception of *Lubricating the lips*, which was planned for 65.1% (Figure 1a). Figure 1b shows that not all older persons with problems related to *Dentures*, *Tongue* and *Mucous membrane* had planned interventions. *Tooth brushing* was the most common planned intervention for problems related to the *Gums* (72.0%) and *Teeth* (67.9%) (Figure 1c,d).

## 5 DISCUSSION

The current study shows that among older persons with assessed oral health problems, a large majority had planned preventive interventions, and approximately three quarters of these interventions were completed. However, there was a mismatch between type of oral health problem and preventive interventions. Common oral health problems were related to the *Teeth*. *Impaired ability to understand information and instructions* was the most common cause of oral health problems, and *Tooth brushing* was the most common planned intervention.

To the best of our knowledge, our study is the first to investigate the correspondence between specific oral health problems and associated planned preventive interventions according to ROAG‐J. While our study focused on correspondence between specific problems and associated interventions, a recent study by Bellander, Andersson, Wijk, et al. (2021) investigated longitudinal effect of preventive interventions according to ROAG‐J and found some improvements in oral health. Our results demonstrate inconsistency between risk and preventive interventions for oral health problems. Only two‐thirds of the older persons with identified risk for poor oral health related to the *Teeth* had *Tooth brushing* as a planned preventive intervention. On the one hand, it could be argued that all older persons would most likely benefit from such care, especially because tooth brushing after each meal and cleaning the dentures once a day can reduce the incidence of aspiration pneumonia among frail older persons (Manger et al., 2017; van der Maarel‐Wierink et al., 2013). On the other hand, this finding may reflect that the older persons brushed their own teeth and were not in need of assistance. Moreover, only 22.5% of the older persons having problems related to the *Tongue* had *Cleaning of the tongue* as a planned intervention, indicating that this intervention needs more emphasis because a recent review (Izumi & Akifusa, 2021) concluded that cleaning the tongue among older persons in nursing homes has a positive influence on swallowing and respiratory functions and prevents aspiration pneumonia. In the total sample, *Cleaning of the tongue* was planned for 7.6%, that is 286 of the older persons, while such intervention was planned for 22.5%, that is 139 of the 618 older persons with oral health problems specifically related to the *Tongue*. This may illustrate an example of a potential mismatch between oral health problems and planned interventions because it could have been expected that a larger proportion with oral health problems specifically related to the *Tongue* had *Cleaning of the tongue* as a planned intervention. Our results thus point towards a need to improve preventive nursing care among older persons about oral health problems. It can be assumed that a better match between type of risk and intervention would decrease the risk for oral health problems and improve quality of life among older persons because previous research has shown such associations (Lindmark et al., 2020; McGrath & Bedi, 1991; van de Rijt et al., 2021) and has shown that the promotion of oral hygiene is an essential basic part in nursing (Pipe et al., 2012).

Because lack of knowledge, education and training in providing oral care have been reported as reasons among nursing staff for not providing oral care for older persons (Wårdh et al., 2012), our results suggest that nursing programs need to clearly emphasize the importance of assisting older persons with their oral health. Moreover, educational interventions for nurses and assistant nurses in community care are vital in order to identify and act on oral health among older persons. Indeed, a wish for education about preventive oral health care has been raised by nursing staff and managers in nursing homes (Neziraj, Andersson, et al., 2021), which is in line with another recent study in which nursing staff expressed a need for more education and training in oral care despite the fact that the nursing staff reported being good at identifying oral health problems among older persons (Andersson & Persenius, 2021). Griffiths et al. (2021) developed an intervention integrating science and practice using a participatory research design for situations when different professionals are involved. One suggestion is to further develop or culturally adapt an existing intervention into a Swedish context and to involve older persons and their next of kin in the development of such an intervention. Another suggestion is to emphasize the importance of oral health basic nursing care in educational programs.

In line with earlier research based on Senior Alert (Rantzow et al., 2017), oral health problems related to the *Teeth* were the most common and were reported in more than 50% of the older persons in the current study. A majority of the older persons in the current study had at least one planned intervention, which also is in line with Rantzow et al. (2017) who reported similar results. In contrast to our results, which showed that *Tooth brushing* was the most common planned intervention (56.2%), this was planned only among 13.5% of the older persons in the study by Rantzow et al. (2017) in which *Moistening of the mouth* was the most common planned intervention. In our study, the proportion of both any and completed preventive interventions was smaller in home health care than in the other types of housing. This may indicate that preventive work is more challenging and requires other routines than in nursing homes where nursing staff are present more constantly.

Older persons' resistance has been raised as an important barrier to performing oral care (Hoben et al., 2017: Willumsen et al., 2012). In our study, resistance to preventive interventions was only reported among 7.5%, which is in line with Rantzow et al. who reported resistance among 4.8%, but contradicting Willumsen et al. (2012) who reported that almost half of the nurses encountered patients resisting help every day. A recent interview study (Koistinen et al., 2021) with older persons in short‐term care showed that they wanted to maintain control over their tooth brushing and that assistance was unthinkable at present but could be considered when not being able to do so themselves. It was also emphasized that many of the older persons had not visited dental care for many years due, for instance, to hospitalizations, declining health, and because the teeth were no longer a priority. Considering that poor oral health is associated with malnutrition (Lindroos et al., 2019; van Lancker et al., 2012) and aspiration pneumonia (van der Maarel‐Wierink et al., 2011) and has an impact on quality of life in older persons (Lindmark et al., 2020; McGrath & Bedi, 1991; van de Rijt et al., 2021), it seems important to offer regular dental care in municipal care. According to the ROAG‐J, a dentist should be consulted and offered when the older persons score a 3 on any assessment, but it is of course important to take the older person's autonomy into consideration because it may be that a dentist appointment is not desired. However, in our study, *Consultation with dentist* when scoring a 3 on any assessment was planned for 9.7% of the older persons, which is somewhat higher than in Rantzow et al. (2017) who reported 5.7%. This could be viewed as alarming and suggesting room for improvement, but another explanation could be that the older persons have simply declined the offered dentist appointment.

### 5.1 Limitations

A limitation is the lack of available medical data and scant background data in Senior Alert, which most likely would have added value to the interpretation of the results. Another weakness is the missing data for the different performed interventions. However, the missing data were handled according to the listwise deletion, that is, in analyses including intervention the complete cases were used (Kang, 2013). Another limitation may be that the included instructions were not fully adhered to by the registration professionals, which may be an explanation for the missing data. Although Senior Alert contains a clear care process, it could be that planned interventions do not reflect what is really being done in practice resulting in both under‐ and over‐reporting. A strength of the current study is that the sample size was rather large and represented older persons receiving different types of municipal care in a whole county in southern Sweden. Another strength may be that the risk assessments were made based on the validated ROAG‐J instrument (Senior Alert, 2021) and that the quality registry Senior Alert has a coverage of 73% (Senior Alert, 2019), which may indicate that the findings are generalizable for older persons receiving municipal care.

## 6 CONCLUSION

A large majority of older persons with assessed oral health problems had at least one planned preventive intervention, and approximately three quarters of these interventions were completed. Importantly, there seemed to be a mismatch between type of oral health problems and preventive interventions as not all older persons had a planned preventive intervention related to their specific oral health problem. Because oral health is an important element in nursing care, the current findings argue that there is room for improvement and suggest the need for educational interventions for nursing staff about the promotion of oral health.

## ETHICAL APPROVAL

The study was ethically approved by the Regional Ethical Review Board, Lund, Sweden (2015‐484).

## Data Availability

The data that support the findings of this study are available on request from the national quality registry Senior Alert. The data are not publicly available due to privacy or ethical restrictions.
